# Unicentric castleman disease located in the left popliteal fossa: a case report

**DOI:** 10.1186/s12891-022-05213-z

**Published:** 2022-03-23

**Authors:** Haijuan Lv, Hongwei Zhao

**Affiliations:** Department of Radiology, The Second Affiliated Hospital of Jiaxing University, No.1518, HuanCheng North Road, Jiaxing, Zhejiang Province China

**Keywords:** Castleman disease, Lower extremity, Case report

## Abstract

**Background:**

Castleman disease (CD) is a lymphoproliferative disease of unknown etiology, it can affect any lymph nodes of the body but rarely affects the popliteal fossa.

**Case presentation:**

We present a 67-year-old woman with touching solitary painless mass in the left popliteal fossa for one week. Imaging showed multiple soft-tissue masses of different sizes in the left popliteal muscle space, the T1 weighted image showed hypointense to isointense, the fat-suppressed T2 weighted images showed subtle hypersignal intermingled with linear of hypointense,and displayed homogeneous contrast enhancement after administration of gadolinium. Complete surgical resection was performed. Pathologically demonstrated plasma cell type CD.

**Conclusion:**

We described a rare case plasma cell type of UCD located in the popliteal fossa which might help to enrich the clinical spectrum of this rare site and unique subtype of UCD. This case illustrates that CD should be considered in the differential diagnosis of every hypervascularity soft tissue tumor in any anatomic location, especially when they occur in the region of lymph node distribution.

## Background

Castleman disease (CD), also known as giant lymph node hyperplasia, was first described by Benjamin Castleman in 1954. It is a lymphoproliferative disease of unknown etiology, which can occur in in any area where lymphoid tissue is normally found, but rarely affects the popliteal fossa. Pathologically, CD is divided into hyaline-vascular, plasma cell variants or mixed type, hyaline-vascular is more common. We report an unusual case of a 67-year-old female with a plasma cell type of an unicentric CD in the left popliteal fossa.

## Case presentation

A 67-year-old female patient was admitted to the hospital for one week after touching solitary painless mass in the left popliteal fossa. Her past medical history had been uneventful, and her family history exhibited no malignancies.

Physical examination: A round soft tissue nodule was touched behind the left knee joint, approximately 3.0 cm in size, with mild tenderness and mobility. Physical examinations of the chest and abdomen were unremarkable.

Laboratory examination: uric acid 488.0 μmol/L (normal reference value 255 ~ 357 μmol/L), other laboratory examinations showed no abnormalities.

**Fig. 1:**
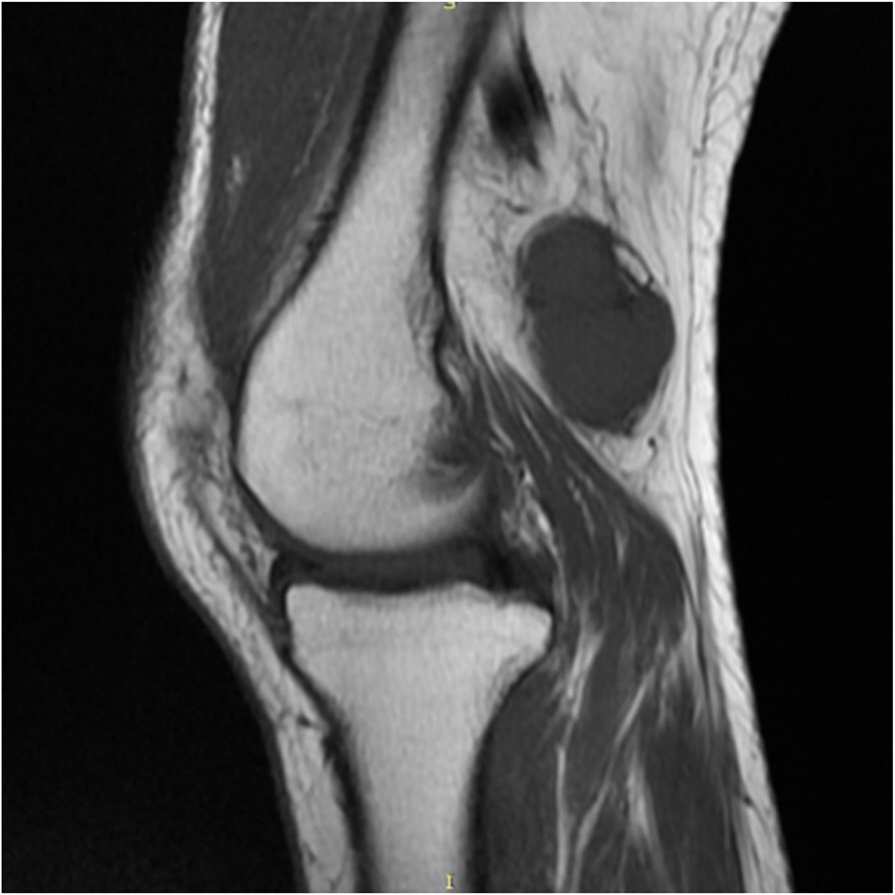
MRI T1 weighted image (sagittal view) showed a hypointense to isointense mass in the left popliteal fossa

**Fig. 2:**
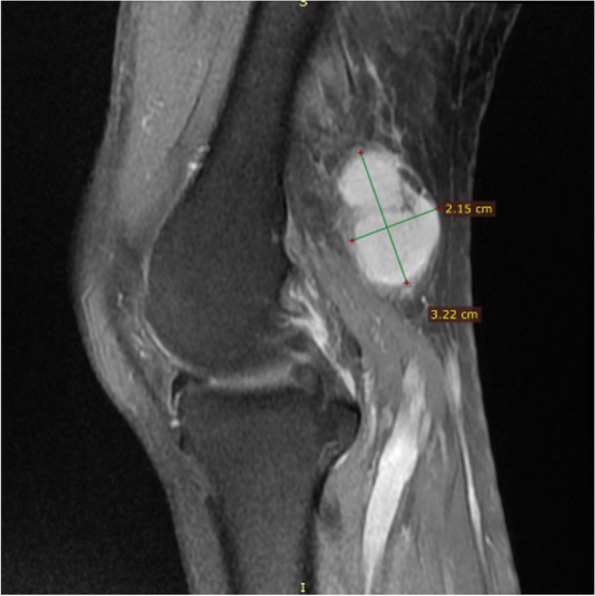
Fat-suppressed T2 weighted image (sagittal view) showed a hyperintense lession,the size was 3.2 cm × 1.9 cm × 2.1 cm

**Fig. 3:**
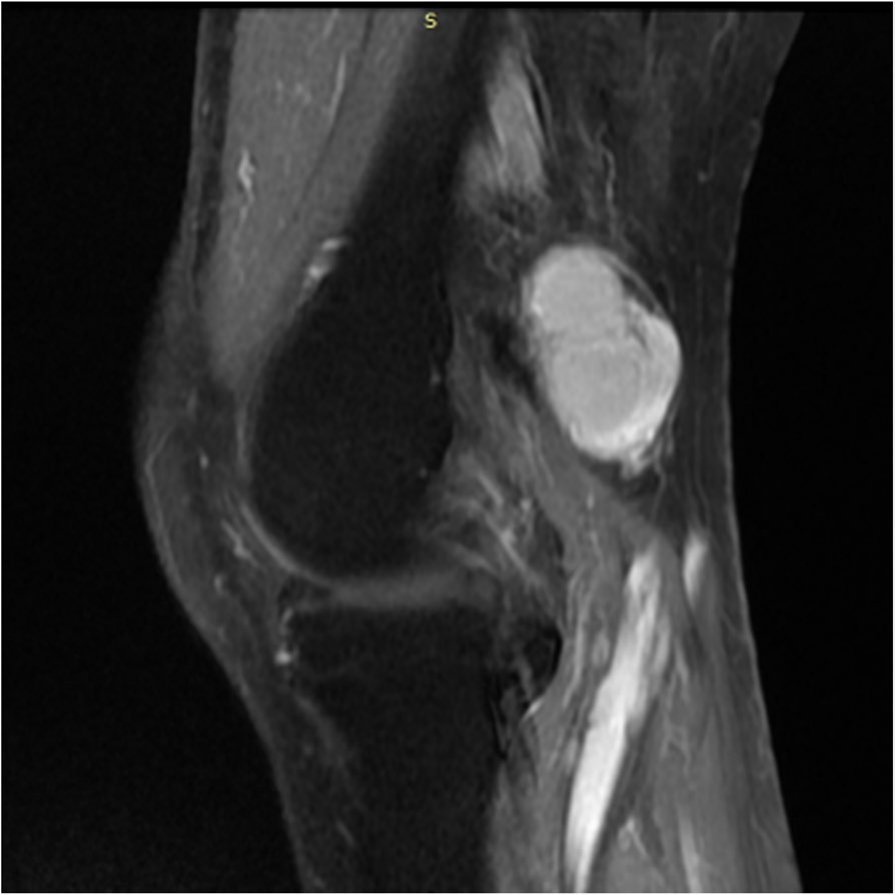
MRI fat-suppressed gadolinium-enhanced T1-weighted image (sagittal and Coronal view) show a sharply demarcated mass with avidly homogeneous enhancement

**Fig. 4:**
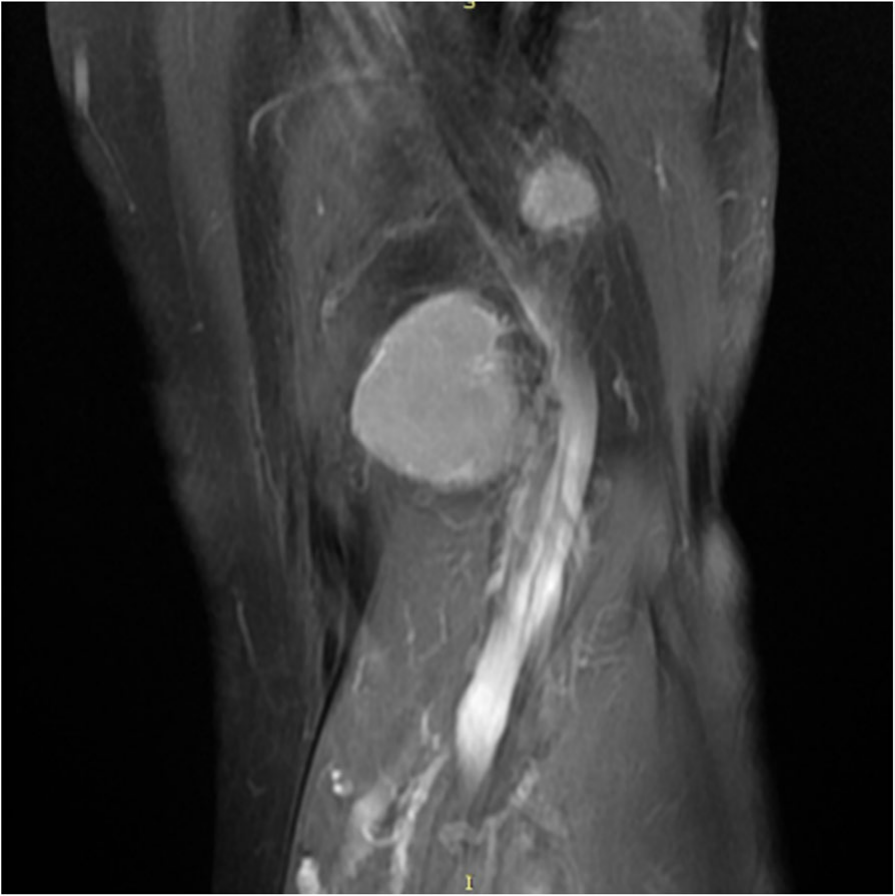
Coronal view shows multiple enhancing soft-tissue mass in the left popliteal fossa

Imaging examination: Ultrasound showed a solid hypoechoic mass of 3.2 cm × 2.4 cm in the left popliteal fossa, with blood flow visible, it was hard to determine whether the lesions are benign or malignant. An unenhanced MRI scan showed multiple soft*-*tissue masses of different sizes in the left popliteal muscle space. The T1 weighted image showed hypointense to isointense, the fat-suppressed T2 weighted images showed subtle hypersignal intermingled with linear of hypointense, and the larger lesion was an oval shape, lesion size was 3.2 cm × 1.9 cm × 2.1 cm, smooth edges, and displayed homogeneous contrast enhancement after administration of gadolinium; two small lesions with similar signal and enhancement degree can be seen in the muscle space above the lesion (Fig. 1, 2, 3 and 4).

Preoperative biopsy revealed a lymphoid tumor of unknown dignity, routine histopathological examination should be performed.

Surgical and pathological findings: Complete surgical resection was performed and three soft nodules having a maximum diameter of 3.2 cm were removed, presented as a sharply demarcated mass lesion. The popliteal arteries and veins were intact.

**Fig. 5:**
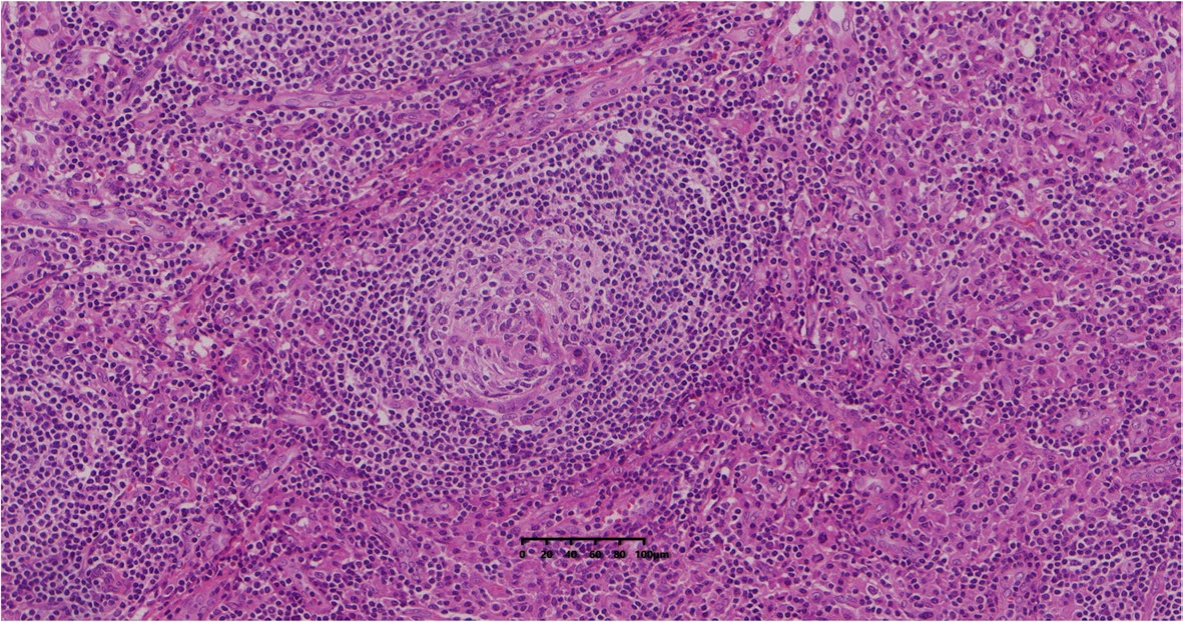
Microscopically, a large number of plasma cell infiltrates were seen in the interfollicular area (H&E, 200 ×)

**Fig. 6:**
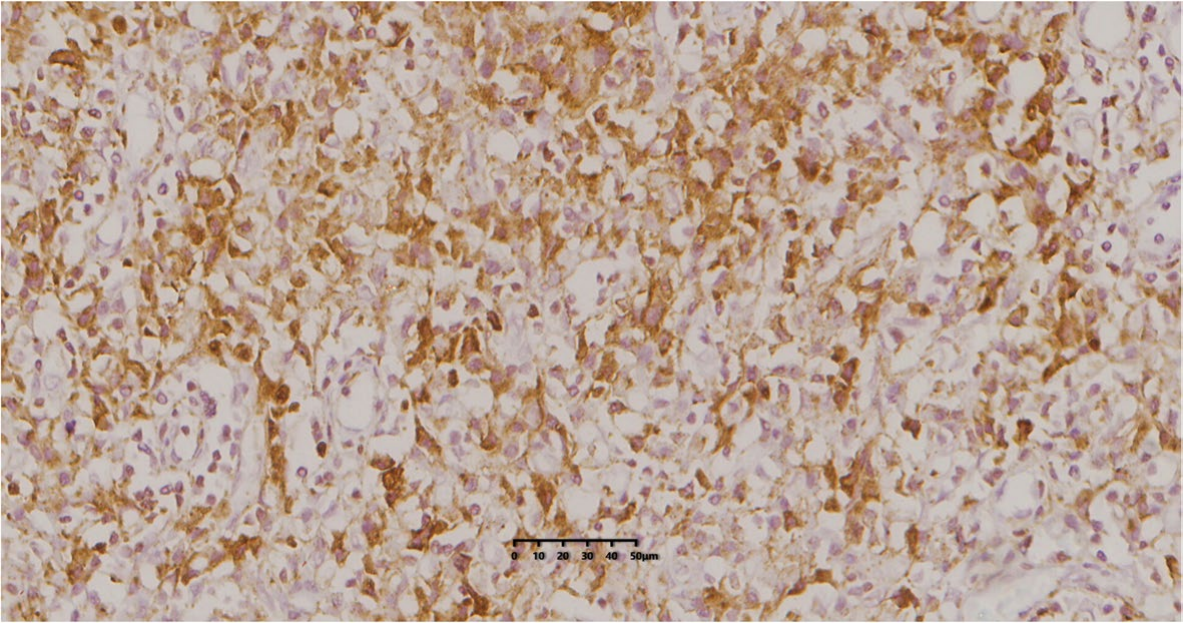
Immunohistochemistry with a primary monoclonal antibody to CD138 shows lymph node infiltration by plasma cells (syndecan-1, 400 ×)

Postoperative pathology: Grossly, the resected tumor specimen displayed a sharply demarcated and medium hardness mass lesion with a gray-red cut surface. Microscopically, the lymph node structure was basically present, the envelope was intact, the lymphatic follicles in the lymph nodes were hyperplastic, the small blood vessels showed varying degrees of hyperplasia, and a large number of patchy, dense plasma cell infiltrates were seen between the follicles (Fig. 5). Immunohistochemical staining: CD3 interfollicular areas ( +), CD43 interfollicular areas ( +), Pax-5 follicular areas ( +), germinal center (Bcl-2-, Ki-67 > 70%), extra-germinal center (Bcl-2 + , Ki-67 about 5%), CD10 germinal center ( +), CD34 vascular ( +), CD21 FDC ( +), plasma cells. CD138 ( +) (Fig. 6), λ ( +) > κ ( +)。The morphological examination was performed using Carl Zeiss Axio Lab.A1. The slides were scanned by using the KF-PRO-005 digital pathology scanner (KFBIO85 company, Ningbo City, China).

Pathological diagnosis: (Multiple left popliteal fossa) Castleman's disease, plasmacytic type.

Postoperative recovery was good and discharged. The patient documented in this report was doing well on 12-month follow-up.

## Discussion and conclusions

Castleman disease (CD) is generally regarded as a benign condition, it is more frequent in women with a median age at diagnosis in the third or fourth decade. Clinically, CD is classified as unicentric CD (UCD) and multicentric CD (MCD) based on anatomical distribution. Unicentric CD tends to be asymptomatic or present with mild symptoms. Multicentric CD can be severely or life-threatening.Recently, a novel clinical classification was mentioned,patients who have more limited lymph node involvement and are referred to as having “regional” or “oligocentric” CD [1].

According to histopathological characteristics, CD can be classified as a hyaline vascular, plasma cell, or mixed type, and the incidence rate is 72%, 18%,and 10%, respectively [2]. UCD affects multiple lymph nodes throughout the body, over 70% of patients with UCD present with the disease in the thorax, with the majority of the cases seen in the mediastinum [3]. Here, we reported an unusual case of UCD located in the lower extremity, an extremely rare site of the disease.

To the best of our knowledge, only a few cases of lower extremity CD have been reported in English literature [2, 4, 5]. Pathological findings for previously reported cases have included hyaline vascular CD and mixed cellularity CD. In the present case, pathologically demonstrated plasmacytic cell type CD, a finding which is rarely reported in the popliteal fossa.

Since the clinical signs and symptoms of UCD are often nonspecific, making them easy to miss or misdiagnose. Lesions in the popliteal fossa require careful evaluation because a number of non-neoplastic and neoplastic lesions can mimic this entity, diagnosing CD without pathological findings is difficult.Generally, the unenhanced CT/MRI scan of CD shows a nonspecific lobulated soft tissue mass, a well-defined border and clearly delineated from adjacent structures, associated with an intact envelope of the lesion.The characteristic image shows that CD is homogeneous enhancement of the lesion, usually without necrolysis liquefaction or hemorrhage, and the hypervascularity soft tissue tumor are associated with the proliferation of small and medium-sized blood vessels in the tissue of the lesion [5]. Although the relatively rare plasmacytic cell type UCD should demonstrate less intense enhancement, however, given the intense lymph node enhancement seen in plasmacytic cell type UCD, it is intuitive that plasmacytic cell type UCD would also avidly enhance [3]. Radiological differential diagnosis of solitary hypervascularity soft tissue tumor includes vascular tumors, extrapleural solitary fibrous tumors, lymphoma, soft tissue sarcoma and metastatic tumor [2].

The guidelines suggest that UCD should be managed in the first-line setting with surgery in both children and adults. Complete surgical excision will usually eliminate any systemic symptomatology and laboratory abnormalities,if present [6]. In cases of unresectable disease, aggressive local therapy with radiation should be considered for patients with symptoms or as consolidation after systemic therapy. Asymptomatic patients may be suitable for observation [7]. Oligocentric CD should be managed more like UCD [1].

This report also has some limitations. First,there was no systemic imaging was performed to look for multicenticity, but notably there were no physical findings suggestive of multicentricity.Second, UCD is virtually always HHV-8 − , but rare positive cases have been reported [6], unfortunately, no relevant viral testing was performed in this case.

In conclusion, we have described a rare case plasmacytic cell type of UCD located in the popliteal fossa which might help to enrich the clinical spectrum of this rare site and unique subtype of UCD. This case illustrates that CD should be considered in the differential diagnosis of every hypervascularity soft tissue tumor in any anatomic location, especially when they occur in the region of lymph node distribution.

## Data Availability

All data generated or analysed during this study are included in this published article。

## Abbreviations

CD: Castleman disease
UCD: Unicentric castleman disease

## References

[1] Rhee FV, Greenway A, Stone K (2018). Treatment of Idiopathic Castleman Disease. Hematol Oncol Clin North Am.

[2] Hakozaki M, Tajino T, Yamada H, Kikuchi S, Hashimoto Y, Konno S (2010). Intramuscular castleman disease of the deltoid: a case report and review of the literature. Skeletal Radiol.

[3] Kligerman SJ, Auerbach A, Franks TJ, Galvin JR (2016). castleman disease of the Thorax: Clinical, Radiologic, and Pathologic Correlation: From the Radiologic Pathology Archives. Radiographics.

[4] Rooney RC, Pitcher JD (1998). castleman diseasein the extremity. Am J Orthop (Belle Mead NJ).

[5] Schaefer IM, Gunnel H, Schweyer S, Korenkov M (2011). Unicentric castleman diseaselocated in the lower extremity: a case report. BMC Cancer..

[6] van Rhee F, Oksenhendler E, Srkalovic G (2020). International evidence-based consensus diagnostic and treatment guidelines for unicentric castleman disease. Blood Adv.

[7] Beckham TH, Yang JC, Chau KW, Noy A, Yahalom J. Excellent Outcomes with Surgery or Radiotherapy in the Management of Castleman Disease Including a Case of Oligocentric Disease. Clin Lymphoma Myeloma Leuk. 2020;20(10):685-9. 10.1016/j.clml.2020.05.002. Epub 2020 May 11. 10.1016/j.clml.2020.05.002 PMC7541423 32522439

